# The equity of China’s emergency medical services from 2010–2014

**DOI:** 10.1186/s12939-016-0507-5

**Published:** 2017-01-11

**Authors:** Ke Yan, Yi Jiang, Jingfu Qiu, Xiaoni Zhong, Yang Wang, Jing Deng, Jingxi Lian, Tingting Wang, Cheng Cao

**Affiliations:** The Research Center for Medicine and Social Development, Collaborative Innovation Center of Social Risks Governance in Health, School of Public Health and Management, Chongqing Medical University, Chongqing, 400016 China

**Keywords:** Emergency medical services, Equity, China

## Abstract

**Background:**

With the depth development of health care system reform in China, emergency medical services (EMS) is confronted with challenges as well as opportunities. This study aimed to analyze the equity of China’s EMS needs, utilization, and resources distribution, and put forward proposal to improve the equity.

**Method:**

Three emergency needs indicators (mortality rate of cardiovascular and cerebrovascular diseases, harm, and digestive system disease), two utilization indicators (emergency outpatient visits and rate of utilization) and one resource allocation indicator (number of EMS facilities) were collected after the review of the China Statistical Yearbook and the National Disease Surveillance System. Next, EMS related indicators were compared among 31 provinces from the eastern, central, and western regions of the country. Concentration Index (CI) were used to measure the equity of EMS needs and utilization among the western, central, and eastern regions. The Gini coefficient of demographic and geographic distribution of facilities represented the equity of resource allocation.

**Result:**

During 2010–2014**,** the CI of cardiovascular and cerebrovascular disease mortality changed from positive to negative, which indicates that the concentrated trend transferred from richer regions to the poorer area. Injury mortality (CI: range from − 0.1241to −0.1504) and digestive disease mortality (CI: range from − 0.1921 to − 0.2279) consistently concentrated in the poorer region, and the inequity among regions became more obviously year-by-year. The utilization of EMS (CI: range from 0.1074 to 0.0824) showed an improvement; however, the inequity reduced gradually. The EMS facilities distribution by population (Gini coefficient: range from 0.0922 to 0.1200) showed high equitability but the EMS facilities distribution by geography (Gini coefficient: range from 0.0922 to 0.1200) suggested a huge gap between regions because the Gini coefficients were greater than 0.5 in the past 5 years.

**Conclusion:**

There are some inequities of needs, utilization, and resource allocation in the China EMS. The government needs to stick to the principle of increasing investment in poorer regions, perfecting ambulance configuration and improving health workers’ professional skills to improve the equity and quality of EMS.

## Background

Due to the rapid elevation of social development, China has witnessed remarkable transformations in the depth population aging, rapid development of industry, and an increasingly sophisticated transportation network. Meanwhile, rapid growth of EMS needs should be taken into consideration carefully [1–4]. According to Chinese cardiovascular and cerebrovascular report 2014, there were 290 million patients with cardiovascular and cerebrovascular diseases, including 270 million hyperpietic and 7 million paralytic. In 2013, rural and urban cardiovascular and cerebrovascular disease mortality rates were respectively 293.69/100,000 and 259.40/100,000. It was estimated that cardiovascular and cerebrovascular diseases accounted for 41% of all deaths in China, which took the lead in all kinds of diseases [5]. Injury has become China’s fourth cause of death, with a death constituent ratio of 8%. According to WHO estimates, injury has become one of the main causes of death in all age groups under 60 years old. The main cause of injury-related death was a lack of access to timely and effective EMS after the accident, especially in poorer regions [6]. Since 1990, digestive system diseases have become one of the top seven causes of death in China. In the report of the death of Chinese residents 2014, the digestive system diseases were the sixth cause of male death and the seventh cause of female death [7]. In the meantime, digestive diseases were always one of the most common diseases in emergency medicine, which consumed large amount of emergency medical resources with a relatively high mortality rate [8].

Rapid and effective emergency work means a lot in terms of saving patients’ lives and reducing the pre-hospital mortality and disability rates. Research suggests that the pre-hospital death rate has changed with medical levels [9]. Timely emergency measures could reduce injury-caused deaths by 80% for adults and 60% for children [10]. It has been noted that “for every woman who dies, at least 30 others are injured and disabled” [11]. An effective job in EMS could save resources for subsequent treatment and reduce both the morbidity and mortality rates [12]. Moreover, studies have shown that patients do not use EMS due mainly to a lack of medical expenses, knowledge, and technical resources [13]. In fact, the distance and transportation costs of EMS can be declined by encouraging the use of ambulance.

The history of emergency medical in China is about 50 years in total, with China’s first formal emergency center built in 1988 as the Beijing Emergency Center. In the next few decades, Chinese people overcame various difficulties such as insufficient investment from the government, an uneven level of medical staff, an insufficient and backward first-class facility, and limited professional skills. Eventually, EMS was established with large and medium–sized cities as the core. As of 2014, China has 325 emergency centers. However, different levels among different regions are restricting the further development of EMS [14]. China has experienced a remarkable disease and death chart transition over the past several years, yet the situation of emergency utilization and resource allocation in poorer regions are still serious issues. EMS has a huge gap between the poorer and richer regions, although the goal of EMS is to provide acute health service to patients in suddenly encountered emergencies [15]. A study that aims to comprehensively analyze the trend of equity in EMS needs, utilization, and resource allocation among different economic regions in China is needed.

## Methods

### Study design

We conducted a descriptive study based on the secondary data collected during 2010–2014. According to the characteristics of emergency medical treatment and availability of data, cardiovascular and cerebrovascular diseases, injury, and digestive system disease mortality rates, which were common in emergency medicine with high mortality, were selected as indicators of EMS needs. The mortality data was collected from the National Disease Surveillance System 2010–2014. Emergency room visits and the number of emergency centers were adopted to reflect the EMS utilization and EMS distribution by consulting the China Health Statistics Yearbook 2010–2014. Geographic area demographics were collected from China Statistical Yearbook 2010–2014.

China can be divided into three distinct regions based on geographical location and economic development level: eastern, central, and western. The eastern regions contains the richer cities, the central region is at a medium level, and the western region contains the poorer cities. The eastern regions include Beijing, Tianjin, Hebei, Liaoning, Shanghai, Jiangsu, Zhejiang, Fujian, Shandong, Shangdong and Hainan; the central regions include Shanxi, Jilin, Heilongjiang, Anhui, Jiangxi, Henan, Hubei and Hunan; and the western regions include Inner Mongolia, Chongqing, Guangxi, Sichuan, Guizhou, Yunnan, Tibet, Shaanxi, Gansu, Qinghai, Ningxia and Xinjiang.

After reviewing and organizing, the data were recorded into Excel and analyzed with SPSS21.0 software. The main analytical methods are descriptive analysis, CI and Gini coefficient.

### Measures of equity evaluation

Generally, health care equity could be judged from horizontal equity and vertical equity. However, there were no uniform standard measures for both of these indicators [16]. Therefore, systematic consideration was conducted on the object of this study and the characteristics of indicators in addition to analysis of whether the EMS between different regions is equitable and how equitable is it by CI and the Gini coefficient.

$$ CI={\displaystyle \sum_{t=1}^T\left({P}_t{L}_{t+1}-{P}_{t+1}{L}_t\right)} $$

In this formula, P represents the cumulative percentage of the population ranked by economic development level, L represents the cumulative percentage of death or emergent patients, and T represents group number. CI values range from − 1 to +1. Negative values suggest that the mortality or EMS utilization is concentrated in poor regions while positive values suggest that the mortality or EMS utilization is concentrated in rich regions. A value of 0 means absolute equity among different regions [17].

**Fig. 1:**
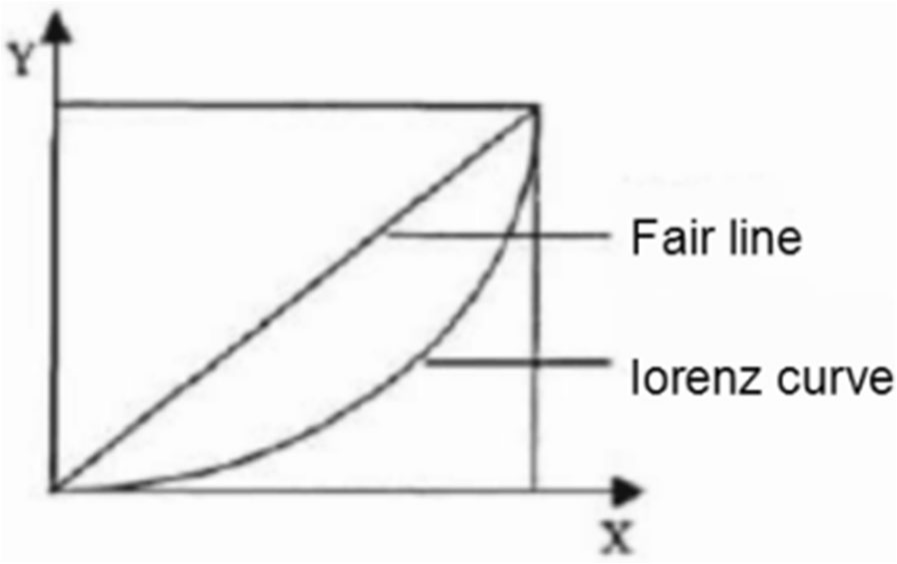
the Lorenz curve

The Gini coefficient was put forward by Hirschmann on the basis of the Lorenz curve, which aimed to judge whether the resource is distributed equally or not [18]. There are two curves in the rectangular coordinate system, one is the fair line and the other is the Lorenz curve. If it is assumed the area between two curves is A and the area under the Lorenz curve is B, then the quotient of A/(A + B) presents the degree of inequity (Fig. 1). This value is known as the Gini or Lorenz coefficient. When the value of A is zero, the Gini coefficient is zero, which suggests complete equity. On the contrary, when the value of B is zero, the Gini coefficient is 1, which suggests complete inequity.

The computation formula is as follows:

$$ {s}_1=\frac{1}{2}{\displaystyle \sum_{i=0}^n\left({Y}_i+{Y}_{i+1}\right)}{X}_{i+1} $$

Gini coefficient =2 × (0.5-S_1_)

## Results

### Equity in EMS needs

**Fig. 2:**
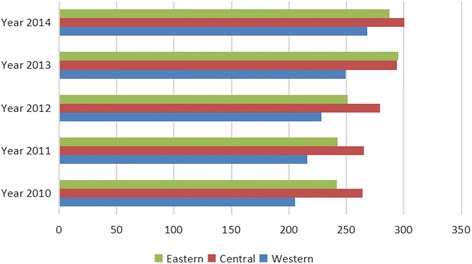
Mortality rate of cardiovascular and cerebrovascular diseases among the different regions, China, 2010 to 2014

**Fig. 3:**
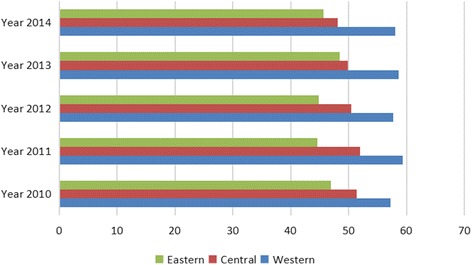
Mortality rate of injury among the different regions, China, 2010 to 2014

**Fig. 4:**
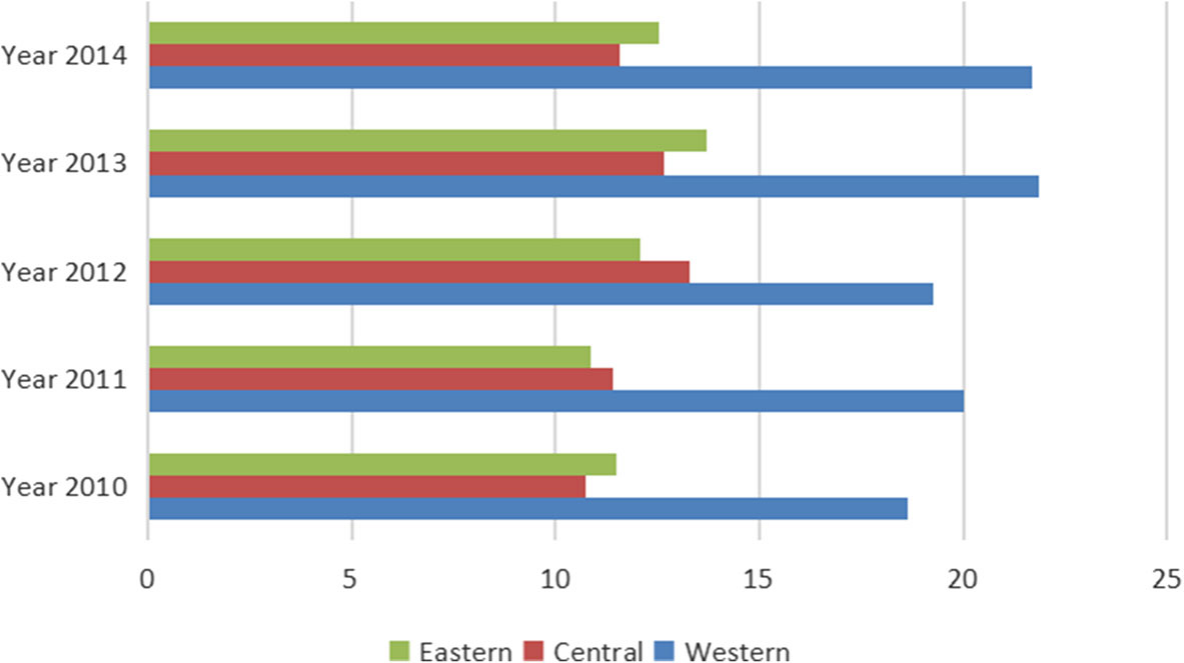
Mortality rate of digestive system disease among the different regions, China, 2010 to 2014

The cardiovascular and cerebrovascular disease mortality rate was fairly high. Moreover, the mortality rate of cardiovascular and cerebrovascular diseases rose by 20.38% over the past 5 years in China, which had the highest rates in the central regions, increased the fastest in the western regions, and the gap among the three different regions declined from 22.30 to 12.02% (Fig. 2). As for injury, the gap was significant. The mortality in the western region was higher than that in the central region, and the mortality in central region was higher than that in the eastern region For example, in 2014, the injury mortality in the eastern, central, and western regions were respectively 45.69 per 100,000 people, 48.14 per 100,000 people, and 58.05 per 100,000 people. In addition, the injury-based mortality increased in the western region and the decrease in the eastern region induced a gap between the regions, which rose from 22.00 to 27.05% (Fig. 3). The mortality rate of digestive system diseases was concentrated in the western region while the mortality rate of the central and eastern regions was at a relatively low level. In 2014, the mortality rates for digestive system diseases were respectively 12.55 per 100,000 people, 11.57 per 100,000 people, and 21.69 per 100,000 people in the eastern, central, and western region. The gap between the three different regions rose from 62.03 to 72.83% in the past 5 years (Fig. 4).

**Fig. 5:**
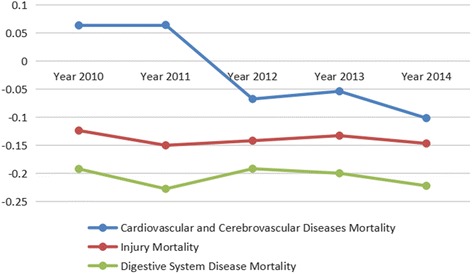
Concentration indicator for each indicator of emergency medical services needs, China, 2010 to 2014

In the past 5 years, the CI of cardiovascular and cerebrovascular diseases changed from a positive value to negative value; the values were 0.0631 in 2010 and −0.1020 in 2014. This change suggests that the concentrated trend of cardiovascular and cerebrovascular diseases mortality transferred from the richer regions to the poorer regions. The CI value of injury mortality and digestive system disease mortality were negative values all the time, respectively − 0.1241– − 0.1504 and −0.1921– − 0.2279; it means the mortality rates of injury and digestive system disease consistently focused on the poorer regions from 2010 to 2014, and fluctuated within a small range. Namely, there were some inequities among the three regions about EMS needs (Fig. 5).

### Equity in EMS utilization

During 2010–2014, emergency room visits presented an increasing trend across the whole country. However, the gap of emergency department use ratio among different regions was obvious. The CI values were 0.1074 in 2010 and 0.0924 in 2014, which were positive and declined year-by-year. This data means that EMS utilization focused on the richer regions while the equity improved slightly (Table 1).

**Table 1:** Concentration indicators, use ratio and emergency room visits among the different regions, China, 2010 to 2014

Year	Western regions		Central regions		Eastern regions		CI
	Emergency outpatient visits (thousand)	Use ratio	Emergency outpatient visits (thousand)	Use ratio	Emergency outpatient visits (thousand)	Use ratio	
2010	20,720	0.0562	15,084	0.0357	43,194	0.0785	0.1074
2011	23,351	0.0645	17,486	0.0413	48,502	0.0875	0.0995
2012	28,459	0.0781	20,295	0.0477	58,652	0.1050	0.0992
2013	31,932	0.0872	23,593	0.0553	64,612	0.1150	0.0924
2014	35,798	0.0972	26,507	0.0619	69,827	0.1235	0.0824

### Equity in EMS facility distribution

Table 2 summarizes the distribution of facilities. Results were as follows: the number of emergency centers in the eastern, central, and western regions increased year-by-year, with the most abundant emergency resources in the eastern region and the least in the western region. When observing the equity related to population, the Gini coefficients were 0.0922–0.1200, which indicated that it was equitable. In contrast, when observing the equity related to geography, the Gini coefficients were 0.5481–0.5696 where 0.5481 was the minimum in 2010 and 0.5696 was the maximum in 2014, which indicated that it was extremely inequitable and the gap was growing bigger and bigger.

**Table 2:** Resources distribution of EMS among the western, central, and eastern regions, China, 2010 ~ 2014

Year		Western regions	Central regions	Eastern regions	Gini
2010	Facilities	55	67	123	
	Population (thousand)	360,700	422,760	550,390	0.0922
	Geographical area (square kilometer)	6878.7	1676.1	1061.7	0.5481
2011	Facilities	58	68	144	
	Population (thousand)	362,220	423,740	554,460	0.1200
	Geographical area (square kilometer)	6878.7	1676.1	1061.7	0.5657
2012	Facilities	62	78	155	
	Population (thousand)	364,280	425,110	558,500	0.1089
	Geographical area (square kilometer)	6878.7	1676.1	1061.7	0.5646
2013	Facilities	65	85	162	
	Population (thousand)	366,370	426,710	562,080	0.1125
	Geographical area (square kilometer)	6878.7	1676.1	1061.7	0.5674
2014	Facilities	66	93	166	
	Population (thousand)	36,839	42,847	56,560	0.1092
	Geographical area (square kilometer)	687.87	167.61	106.17	0.5696

## Discussion

The cardiovascular and cerebrovascular disease mortality rate increased significantly in the past 5 years. The good news is, despite high mortality in all regions, the gaps among regions became smaller and smaller. Guidelines for the primary prevention of stroke showed that changes in diet with an increased intake of total calories, fat, meat and sugar-sweetened beverages is the main determinant of high cardiovascular and cerebrovascular disease levels in China [19]. In recent years, China’s rapid economic development, especially in the western region, has led to the great changes in diet.

The gap of injury and digestive system disease mortality among different regions was evident; the poorer western region continued to have a higher mortality rate than the richer central and eastern regions.

Road injury was identified as a leading cause of premature death in China. The research on the road traffic injury in China from Ma S, et al. showed that most road injury deaths were among pedestrians and motorcyclists [20]. Meanwhile, a large part of residents used a motorcycle as the main method of travel in the poorer western region. In developed countries, deaths caused by digestive diseases were infrequent [21]. However, in China, especially in the poorer regions, the acute gastrointestinal tract and infectious diseases induced by unclean food were common.

EMS utilization in the eastern region was better than that in the western and central regions. Moreover, EMS facility distribution by geography showed extreme inequity the Gini coefficient, which was overall higher than 0.5. Namely, the western and central regions were demonstrated high needs, low utilization, and low offer situations. However, the starting point of EMS fundamental should fit the needs of people. Whether China’s EMS has met patients’ needs and how to distribute EMS resources reasonably should be taken into consideration.

Unlike other medical resources, EMS requires a small service radius and emergency response time [22–24]. Of the areas with a relatively perfect emergency medical system, the emergency response time is 4 to 6 minutes in the United States, 4 minutes in Japan, and 7 to 10 minutes in Germany. As for China, the emergency response time is 12 min in Beijing, 11 min in Shanghai, and 12 min in Guangzhou. In accordance with the requirement of the National Health and Family Planning Commission of PRC, the service radius of an emergency medical sub-station is about three to five kilometers, and is decreased to some extent in the densely populated areas [25]. In view of the high cardiovascular and cerebrovascular disease mortality rates and high growth rate in the nation as a whole, an emergency station is supposed to set very three kilometers in richer regions. Ordinarily, an emergency station is supposed to set every five kilometers. For remote, mountainous regions, problems of longer emergency response time could be made up through the popularity of CPR and other basic first-aid skills [26], and the training of mutual aid consciousness of the masses. In fact, the popularity of emergency knowledge should be not only implemented in remote areas, but also across the whole nation. After breathing and the heartbeat stop, the mortality rate increases by 10% for every 1 minute delayed. In 2012, Shanghai started to collocate automated external defibrillator in schools and subway stations; such a policy should be promoted nationwide.

The quality of pre-hospital emergency care can affect the outcome of the patient’s condition radically. When the emergency ambulance is equipped with an experienced physician, completed rescue equipment, and medicine, effective treatment can be provided for critically ill patients. After the vital signs become stable, the patients can be transferred to the relevant department or intensive care unit directly. According to the regional death spectrum, rescue equipment and medicine should be adapted to suit the local conditions. Through the analysis of a certain hospital’s 3210 emergency visits, Li Dengkai found that most visits only played the role of transition. Highly trained medical staff were often only given a simple dressing and bleeding and external fixation process, which is a huge waste from a health economics point of view [27]. With the depth of medical reform, ambulance configuration has been improved. However, in poorer regions, shortage of drugs and equipment in ambulance emergencies still limits the ambulance’s ability to play a greater role. In view of the above circumstances for the poorer regions, the ambulance should be equipped with not only cross box, outer box, medical oxygen cylinders, cars stretcher, circulatory system emergency kit, emergency medicine, and vehicle-carried communication system, but also with ventilator, suction, a portable electrocardiogram machine, surgical trauma emergency kit, debridement package, gastrointestinal decompression package, antibiotics, antidotes, analgesics, local anesthetics, and hormone drugs in the western region, and ventilator, ECG monitor, automatic defibrillator, vasopressors, antihypertensive drugs, cardiac drugs, antiarrhythmic drugs, vasodilator, diuretic dehydration drugs and tracheotomy package in the central region [28].

With the increasing demand for EMS needs, the lack of emergency physicians recently became one of the top dilemmas. Compared to moderately developed countries that have one emergency doctor per 10,000 people, China has one emergency doctor per 110,000 people. By 2020, the shortfall of emergency physicians will increase to 131,800. The emergency physician shortage is a burning problem [29]. In order to alleviate the current difficulties, the state implemented an additional extra marks policy in 2015 for emergency physicians in examination of medical practitioners, but this implementation is far from enough. The training and cultivation of emergency medical technicians must be improved immediately, especially in the poorer regions [30, 31]. This growth can be achieved through the following four points after studying China’s health care reform. First, in reference to Chinese rural doctor training mode with funding support from the financial sector, enrollment targets from education departments, and training resources from medical colleges to establish an emergency physician orientation cultivation [32–34]. Second, in consideration of the actual characteristics of emergency medical work, the 3-year resident standardization training should be shortened to 2 years with the residents proceeding emergency professional training in the third year [35]. Third, favored promotion and treatment policies for emergency doctors should be formulated. Emergency medical performance bonuses should not be lower than the average level of the various departments of the hospital [32, 36]. Lastly, the health administrative departments should provide an emergency medical outflow policy to address emergency medical work problems brought on by the increase of age in consideration of the work stress in the emergency department is too high, and emergency physicians over the age of 40 can choose whether to go to the small intensity work department, which can reduce worries for emergency doctors.

### Study limitations

There are several main limitations in this study. First, the EMS-related data in this study are derived from the Health Statistics Yearbook. The sample errors and non-sample errors in the Health Statistical Yearbook also apply this. For example, the cause of death in an out-of-hospital death case may not be accurately accounted for. Second, gender equity is an important component in health services analysis, but due to the limitations of data sources, we have not been able to obtain data on emergency medical services for patients of different genders, so this study is not able to compare the equity of emergency medical services from the gender perspective. Third, from 2010 to 2012, the National Disease Surveillance System reported the mortality of cardiovascular and cerebrovascular diseases. From 2013 to 2014, the mortality rates of heart disease and cerebrovascular disease were reported separately. In this case, the death rate of heart disease and cerebrovascular disease were added together to represent the mortality of cardiovascular and cerebrovascular diseases in that year.

## Conclusion

In China, EMS is inequitable among the western, central, and eastern regions. The inequity of EMS provisions and utilization led to the inequity of EMS needs. The government should attach more importance to the possible policy mentioned above to narrow the gap among different regions. The rescuing radius should be shortened as much as possible according to the geographical factors and economic conditions, ambulance configuration based on local death spectrum must be improved, and the training of health personnel must be strengthened, especially in poorer regions.

## Abbreviations

CI: Concentration index
EMS: Emergency medical services
